# SSRIs differentially modulate the effects of pro-inflammatory stimulation on hippocampal plasticity and memory via sigma 1 receptors and neurosteroids

**DOI:** 10.1038/s41398-023-02343-3

**Published:** 2023-02-03

**Authors:** Yukitoshi Izumi, Angela M. Reiersen, Eric J. Lenze, Steven J. Mennerick, Charles F. Zorumski

**Affiliations:** 1 Department of Psychiatry & Taylor Family Institute for Innovative Psychiatric Research, Washington University School of Medicine, St. Louis, MO USA; 2 Center for Brain Research in Mood Disorders, Washington University School of Medicine, St. Louis, MO USA

**Keywords:** Hippocampus, Depression

## Abstract

Certain selective serotonin reuptake inhibitors (SSRIs) have anti-inflammatory effects in preclinical models, and recent clinical studies suggest that fluvoxamine can prevent deterioration in patients with COVID-19, possibly through activating sigma 1 receptors (S1Rs). Here we examined potential mechanisms contributing to these effects of fluvoxamine and other SSRIs using a well-characterized model of pro-inflammatory stress in rat hippocampal slices. When hippocampal slices are exposed acutely to lipopolysaccharide (LPS), a strong pro-inflammatory stimulus, basal synaptic transmission in the CA1 region remains intact, but induction of long-term potentiation (LTP), a form of synaptic plasticity thought to contribute to learning and memory, is completely disrupted. Administration of low micromolar concentrations of fluvoxamine and fluoxetine prior to and during LPS administration overcame this LTP inhibition. Effects of fluvoxamine required both activation of S1Rs and local synthesis of 5-alpha reduced neurosteroids. In contrast, the effects of fluoxetine did not involve S1Rs but required neurosteroid production. The ability of fluvoxamine to modulate LTP and neurosteroid production was mimicked by a selective S1R agonist. Additionally, fluvoxamine and fluoxetine prevented learning impairments induced by LPS in vivo. Sertraline differed from the other SSRIs in blocking LTP in control slices likely via S1R inverse agonism. These results provide strong support for the hypothesis that S1Rs and neurosteroids play key roles in the anti-inflammatory effects of certain SSRIs and that these SSRIs could be beneficial in disorders involving inflammatory stress including psychiatric and neurodegenerative illnesses.

## Introduction

Selective serotonin reuptake inhibitors (SSRIs) have been mainstays of psychopharmacology for over 30 years with beneficial effects in major depressive disorder (MDD), anxiety disorders and complex syndromes such as obsessive-compulsive disorder (OCD) [1]. Effects in psychiatric illnesses are thought to reflect, at least in part, well-known effects on serotonin transporters (SERTs) and changes in serotonin levels in innervated regions. Nonetheless, SSRIs also have actions independent of serotonin that could contribute to therapeutic efficacy. These latter effects include modulation of neuroinflammation, autophagy, and intracellular stress [1, 2].

SSRIs are lipophilic weak bases and readily access sites within cell membranes and intracellular compartments including direct effects on endoplasmic reticula (ER), Golgi, lysosomes, and the NLRP3 inflammasome, among others [3–6]. Potential non-serotonin targets include sigma-1 receptors (S1Rs), acid sphingomyelinase (ASM), and cellular enzymes involved in the synthesis of neurosteroids from cholesterol [7–13]. These various effects can promote intracellular production of brain-derived neurotrophic factor (BDNF) and activation of its primary receptor, tropomyosin receptor kinase B (TrkB receptors), a mechanism thought to contribute to antidepressant effects [14]. Certain SSRIs also appear to interact directly with TrkB receptors [15].

Among non-SERT targets of SSRIs, S1Rs are intriguing because of the important role that these receptors play in modulating ER stress responses, mitochondrial function, inflammation, and neurosteroidogenesis [16–22]. Several SSRIs interact directly with S1Rs serving as agonizts in the case of fluvoxamine and fluoxetine, and antagonists or inverse agonists in the case of sertraline [8, 23]. Among SSRIs, fluvoxamine is the most potent S1R ligand, acting at concentrations readily achieved with therapeutic use and only about 10-fold higher than effects on SERTs [8, 23, 24]. A positron emission tomography (PET) study found that a single dose of fluvoxamine (150-200 mg) occupied about 60% of S1Rs in the human brain [25]. In animal models of sepsis, the S1R agonist actions of fluvoxamine dampen inflammation and promote survival [26]. There is also evidence that fluvoxamine and fluoxetine can potentiate the effects of nerve growth factor (NGF) via S1Rs [8]. These preclinical results prompted subsequent human clinical trials of fluvoxamine as a treatment to prevent clinical deterioration in patients with the new onset of COVID-19 [27–29].

The recent human trials in COVID-19, where inflammation is a major contributor to clinical deterioration, prompted us to examine the effects of fluvoxamine and other SSRIs in a model of acute hippocampal dysfunction resulting from exposure to lipopolysaccharide (LPS). LPS is a bacterial wall endotoxin and pro-inflammatory stimulus that impairs synaptic plasticity via the activation of microglia [30–32]. Here we examined the hypothesis that effects on S1Rs play a key role in acute anti-inflammatory actions of specific SSRIs. We tested this hypothesis using acute pre-treatment with SSRIs prior to the delivery of a strong pro-inflammatory stimulus. These experiments were conducted in ex vivo hippocampal slices because of the well-characterized actions of LPS on synaptic plasticity, the high degree of control over drug administration, and the known biology of this preparation [30–32].

## Materials and methods

### Hippocampal slice preparation

Protocols for animal experiments were approved by the Washington University IACUC. Hippocampal slices were prepared from randomly selected postnatal day (P) 28-33 Harlan Sprague-Dawley male albino rats (Indianapolis IN) using published methods [33, 34]. For slice preparation, rats were anesthetized with isoflurane, and dissected hippocampi were pinned on an agar base in artificial cerebrospinal fluid (ACSF) containing (in mM): 124 NaCl, 5 KCl, 2 MgSO_4_, 2 CaCl_2_, 1.25 NaH_2_PO_4_, 22 NaHCO_3_, 10 glucose, gassed with 95% O_2_–5% CO_2_ at 4-6^o^C. The dorsal two-thirds of the hippocampus was cut into 500 µm slices using a rotary slicer and maintained in ACSF at 30 °C for at least 1 hour before experiments.

### Hippocampal slice physiology

At the time of the study, single slices were transferred to a submersion-recording chamber and perfused with 30 °C ACSF at 2 ml/min. Extracellular recordings were obtained from the apical dendritic region of area CA1 to monitor field excitatory postsynaptic potentials (EPSPs) by an experimenter who was not blinded to conditions. EPSPs were evoked using 0.1 ms constant current pulses to the Schaffer collateral pathway once per minute via a bipolar stimulating electrode. Stimulus intensity during experiments was half-maximal based on control input-output (IO) curves. All animals were included in analyses unless baseline recordings were unstable. LTP was induced using a single 100 Hz by 1 s high-frequency stimulation (HFS) of the Schaffer collateral pathway. IO curves were repeated 60 min following HFS and were the primary measure of synaptic change in comparison to baseline (pre-HFS). For display in figures, responses are typically shown at 5 min intervals.

### Behavioral studies

We examined the acute effects of SSRIs on LPS-induced memory impairment using a one-trial inhibitory avoidance learning task that has previously been linked to CA1 hippocampal LTP [31, 34, 35]. In this task, randomly selected P28–33 male rats are placed in an apparatus that has two chambers. One chamber is lit and the other is dark. Both compartments have a floor of stainless steel rods (4 mm diameter, spaced 10 mm apart) through which an electrical shock can be administered in the dark chamber. The safe (lit) compartment is illuminated by four 13 W lights with a light intensity of 1000 lux; the light intensity in the dark chamber was <10 lux. On the first day of experiments, rats were placed in the lit chamber and allowed to habituate to the apparatus by freely moving between chambers for 10 min. On the second day, rats were administered vehicle (DMSO), fluvoxamine (10 mg/kg i.p.), or fluoxetine (10 mg/kg i.p.). One hour later, animals received a single injection of LPS (1 mg/kg i.p.) or saline 1 h prior to conditioning [31]. At the time of training, animals were initially placed in the lit compartment and allowed to explore the apparatus freely for up to 300 s (5 min). When the rats completely entered the dark chamber, they were administered a single-foot shock. Upon returning to the lit chamber, animals were removed from the apparatus and returned to their home cages. On the third day of the experiment, rats were placed in the lit chamber without any drug treatment and the time spent in the lit chamber was recorded over a 300 s trials.

### Chemicals

Salts and fluvoxamine (CAS#:61718-82-9), sertraline (CAS#:79559-97-0), PRE-084 (CAS#:138847-85-5), dutasteride (CAS#:164656-23-9) and LPS were purchased from Millipore Sigma (St. Louis MO). Fluoxetine (CAS#:56296-78-7) and NE-100 (CAS#:149409-57-4) were purchased from Tocris Bioscience (Ellisville MO). Finasteride (CAS#:98319-26-7) was purchased from Steraloids (Newport RI).

### Data collection and analysis

**Fig. 1:**
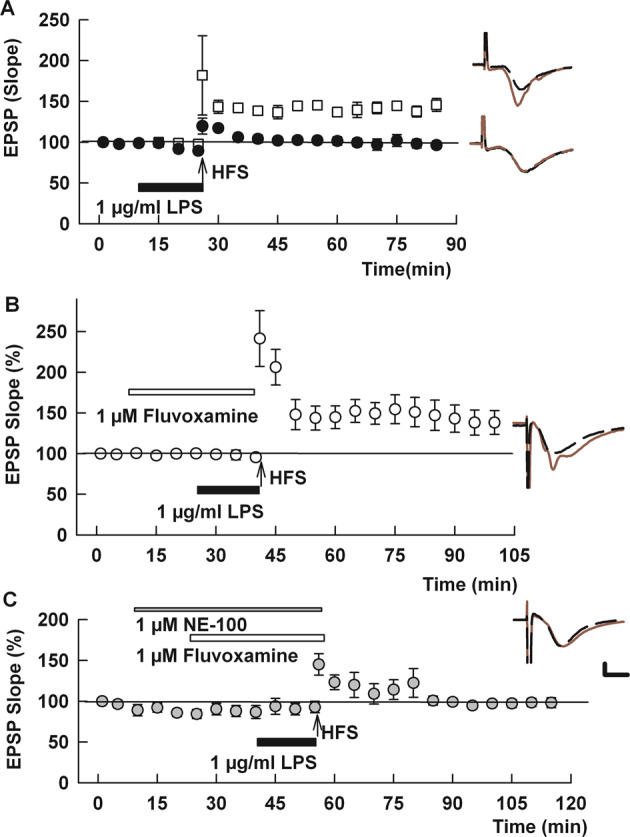
Fluvoxamine overcomes LPS-induced LTP inhibition. **A** In control hippocampal slices, a single 100 Hz × 1 s HFS (arrow) readily induces LTP (white squares). Fifteen-minute administration of 1 μg/ml LPS (black bar) just prior to HFS completely inhibits LTP (black circles). **B** When administered for 30 min prior to and during LPS, fluvoxamine (white bar) prevented the effects of LPS on LTP. **C** NE-100 (gray bar), a selective S1R antagonist, overcame the effect of fluvoxamine on LPS-mediated LTP block. Statistical analyses for this and other figures are provided in the text and Supplemental Table 1. Traces to the right of the graphs in this and all figures show representative EPSPs at baseline (dashed traces) and 60 in the following HFS (solid red traces). Calibration: 1 mv, 5 ms.

**Fig. 2:**
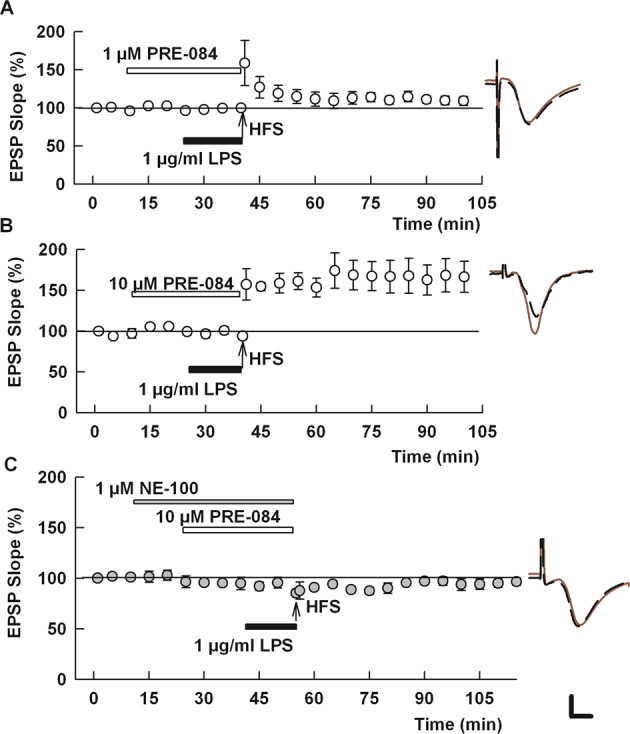
A selective S1R agonist overcomes the effects of LPS on LTP. **A**, **B** At 1 μM, PRE-084 had weak effects on LPS-mediated LTP block (**A**), but 10 μM PRE-084 completely overcame the effects of LPS (**B**). **C** The effects of PRE-084 were blocked by N-100, an S1R antagonist [36]. Traces show representative EPSPs. Calibration: 1 mV, 5 ms.

**Fig. 3:**
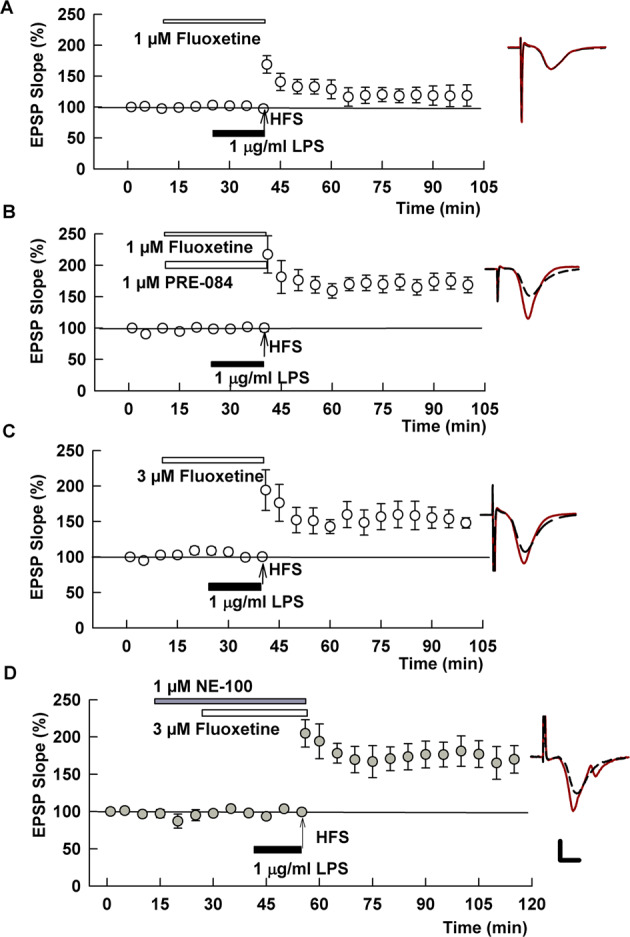
The SSRI, fluoxetine, overcomes the effects of LPS on LTP. **A** When administered at 1 μM, fluoxetine had partial, but non-significant effects on LPS-induced LTP inhibition. **B** A combination of 1 μM fluoxetine with 1 μM PRE-084 completely overcame LPS. **C** Results in panel C prompted an examination of a higher concentration of fluoxetine. At 3 μM, fluoxetine also completely overcame LPS. **D** Unlike fluvoxamine, the S1R antagonist NE-100 had no effect on the ability of fluoxetine to overcome LPS. Traces show representative EPSPs. Calibration: 1 mV, 5 ms.

**Fig. 4:**
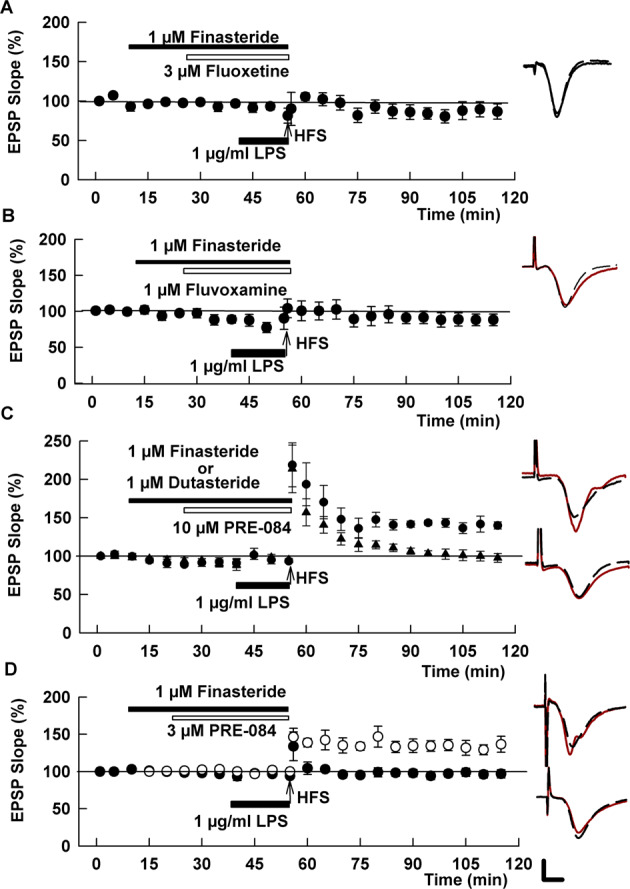
Effects of fluoxetine and fluvoxamine on LPS require synthesis of 5-alpha reduced neurosteroids. **A** The 5AR antagonist, finasteride, which inhibits the synthesis of 5-alpha-reduced neurosteroids, completely blocked the effects of fluoxetine on LPS. **B** Similar to fluoxetine, finasteride also overcame the effects of fluvoxamine on LPS. **C** In contrast to the SSRIs, finasteride did not overcome the effects of the S1R agonist, PRE-084, on LPS (black circles). However, a broader spectrum 5AR inhibitor, dutasteride (black triangles), prevented the ability of PRE-084 to promote LTP in the presence of LPS. **D** An intermediate concentration of PRE-084 (3 μM, white circles)) overcame the effects of LPS and was reversed by finasteride (black circles), akin to the SSRIs. Traces show representative EPSPs. Calibration: 1 mV, 5 ms.

**Fig. 5:**
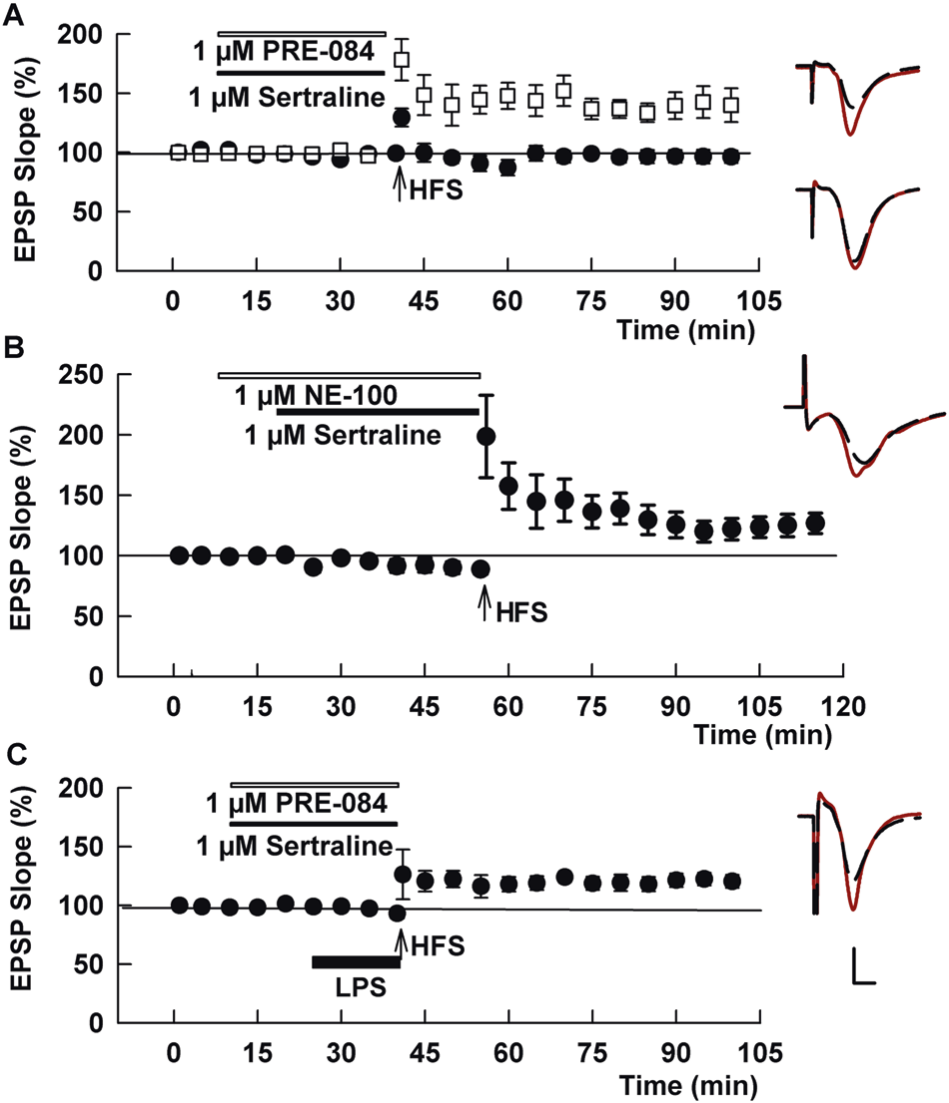
The SSRI sertraline acutely inhibits LTP in contrast to fluvoxamine and fluoxetine. **A** At 1 μM, sertraline blocked the ability of a single HFS to induce LTP (black circles). This LTP inhibition was overcome by the S1R antagonist, PRE-084 (white squares). **B** The S1R antagonist, NE-100 also overcame LTP inhibition by sertraline. **C** A combination of sertraline plus PRE-084 also allowed LTP in the presence of LPS. Traces show representative EPSPs. Calibration: 1 mV, 5 ms.

Experiments were performed and analyzed using pClamp software (Molecular Devices, Union City CA). Results in the text are expressed as mean ± SEM and physiological results are based on analysis of IO curves obtained at baseline and 60 min following HFS. EPSPs were normalized to baseline recordings (taken as 100%). Statistical comparisons were based on IO curves at baseline and 60 min following HFS based on changes in the maximal rising slope of EPSPs evoked by 50% maximal stimuli, with *p* < 0.05 considered significant. A two-tailed unpaired Student’s *t*-test was used for comparisons between groups in all physiological studies (Figs. 1–5 and Supplemental Figs. 1–3). Behavioral studies with multiple comparisons were analyzed using Dunnett’s multiple comparison test. Numbers reported in the text are the number (*N*) of animals studied in a condition. Based on our prior experience using hippocampal slices to study synaptic plasticity and one-trial learning to study memory formation in vivo, these experiments were designed for *N*’s of 5–8. Statistics for physiological studies were performed using commercial software (SigmaStat, Systat Software, Inc., Richmond City, CA). For behavioral studies, Dunnett’s multiple comparison tests was performed using commercial software (GraphPad Prism 9.2.0, GraphPad Software, La Jolla California). Details of statistical analyses for physiological experiments in Figs. 1–5 is provided in Supplemental Table 1. Effect sizes were estimated using Hedge’s *g*. Data in figures display continuous monitoring of responses at low frequency and thus may differ from numerical results described in the text, which are based on analysis of IO curves.

## Results

As we showed previously [31], 1 μg/ml LPS administered for 15 min prior to delivery of a single 100 Hz × 1 s HFS inhibited induction of LTP in the CA1 region of rat hippocampal slices (control LTP: 136.0 ± 5.7% (*N* = 5) of baseline EPSPs measured 60 min following HFS vs. HFS + LPS: 97.6 ± 4.7%, *N* = 5; *p* < 0.001; Fig. 1A; Supplemental Table 1). This LTP inhibition results from strong pro-inflammatory stimulation that activates microglia and signaling via canonical and non-canonical inflammatory pathways [31]. Based on the ability of fluvoxamine to dampen inflammatory and cellular stress mechanisms [12, 26], we examined whether fluvoxamine would alter the effects of LPS on synaptic plasticity. When administered for 30 min prior to HFS, 1 μM fluvoxamine alone had no effect on induction of LTP (136.1 ± 6.0% of baseline 60 min following HFS, *N* = 5, *p* = 0.99 vs. control LTP; Supplemental Fig. 1A). However, when fluvoxamine was administered for 30 min before and during LPS application, LTP was readily induced by HFS (148.8 ± 10.6% of baseline, *N* = 6, *p* < 0.01 vs. LPS alone and *p* = 0.35 vs. fluvoxamine alone; Fig. 1B).

Fluvoxamine can dampen inflammatory responses through the activation of S1Rs [12, 25]. To examine the role of S1Rs in the effects of fluvoxamine on plasticity, we used the selective S1R antagonist, NE-100 [36]. When administered at 1 μM, NE-100 alone had no effect on the ability of HFS to induce LTP (141.3 ± 9.2%, *N* = 5, *p* = 0.64 vs. control LTP; Supplemental Fig. 2). Similarly, NE-100 in combination with 1 μM fluvoxamine had no effect on LTP induction (138.9 ± 6.9%, *N* = 5; *p* = 0.84 vs. LTP with NE-100 alone, Supplemental Fig. 2). In contrast, NE-100 completely reversed the effects of fluvoxamine on LPS-mediated LTP inhibition, resulting in LTP inhibition in the presence of fluvoxamine plus 1 μM NE-100 and LPS (97.7 ± 5.5% of baseline, *N* = 6, *p* < 0.01 vs. LPS + fluvoxamine; Fig. 1C).

Results with fluvoxamine prompted us to examine the effects of a more selective, non-SSRI S1R agonist, PRE-084 [37]. PRE-084 alone had no effect on LTP induction in naïve slices (142.4 ± 14.7% of baseline, *N* = 5, *p* = 0.70 vs. control LTP; Supplemental Fig. 2). At 1 μM, PRE-084 marginally overcame the block of LTP by LPS (114.7 ± 5.9%, *N* = 6, *p* = 0.054 vs. LPS alone; Fig. 3A). A higher concentration of PRE-084, 10 μM, completely overcame the effects of LPS (147.7 ± 10.0%, *N* = 5, *p* < 0.005 vs. LPS alone; Fig. 3B). The ability of 10 μM PRE-084 to overcome the effects of LPS was blocked by the S1R antagonist, NE-100 (92.8 ± 2.6%, *N* = 5; *p* < 0.001 vs. PRE-084 + LPS; Fig. 3C).

We next examined a second SSRI, fluoxetine, an agonist of S1Rs with lower potency than fluvoxamine [8, 23]. Administered alone, fluoxetine had no effect on LTP induction (142.7 ± 8.5%, *N* = 5, *p* = 0.53 vs. control LTP; Supplemental Fig. 1B). At 1 μM, fluoxetine had variable but non-significant effects on LTP inhibition by LPS (126.4 ± 16.3% of baseline, *N* = 5, *p* = 0.13 vs. LPS alone; Fig. 3A). However, when administered with 1 μM PRE-084 in the presence of LPS we observed robust LTP induction (171.8 ± 11.0%, *N* = 5, *p* < 0.001 vs. LPS alone; Fig. 3B). This latter observation coupled with the lower potency of fluoxetine at S1Rs prompted us to examine a higher concentration of fluoxetine. When administered at 3 μM, fluoxetine completely overcame the effects of LPS on LTP (150.0 ± 13.8% of baseline, *N* = 5; *p* < 0.01 vs. LPS alone; Fig. 3C). Unlike fluvoxamine, however, the effects of the higher concentration of fluoxetine were not altered by NE-100 (155.9 ± 6.9%, *N* = 5, *p* = 0.71 vs. 3 μM fluoxetine + LPS; Fig. 3D).

In addition to its effects on serotonin transporters and S1Rs, fluoxetine promotes the synthesis of endogenous 5α-reduced GABAergic neurosteroids including allopregnanolone [13, 37], which in turn modulates LTP [32, 33]. To examine the role of 5α-reduced neurosteroids in SSRI effects, we used the 5α reductase (5AR) inhibitor, finasteride. In prior studies, we found that 1 μM finasteride blocks stressor-induced immunostaining for 5α-reduced neurosteroids in the CA1 region and inhibits modulation of LTP by endogenous neurosteroids and certain pharmacological agents that act via these neurosteroids [32, 33]. At 1 μM, finasteride had no effect on LTP in control slices (130.8 ± 4.3%, *N* = 5, *p* = 0.49 vs. control LTP, Supplemental Fig. 3) but completely prevented the effects of 3 μM fluoxetine on LPS-mediated LTP inhibition (89.3 ± 5.5%, *N* = 5, *p* < 0.005 vs. fluoxetine + LPS; Fig. 4A).

Results with fluoxetine prompted us to examine finasteride against fluvoxamine, based on prior studies indicating that fluvoxamine can also promote endogenous neurosteroid synthesis [13, 38, 39]. Like fluoxetine, finasteride prevented the effects of fluvoxamine on LPS-mediated LTP inhibition (93.6 ± 6.1%, *N* = 5, *p* < 0.005 vs. fluvoxamine + LPS; Fig. 4B). Because S1Rs promote neurosteroidogenesis [17, 20, 21], we also examined effects of finasteride on 10 μM PRE-084 and found that the 5AR inhibitor had no effect on the ability of the S1R agonist to overcome LPS-mediated LTP block, in contrast to effects on the two SSRIs (141.2 ± 7.9%, *N* = 5, *p* = 0.63 vs. PRE-084 + LPS, Fig. 4C). Finasteride has greater potency at Type II 5ARs, and hippocampus also expresses Type I 5AR [40, 41]. Thus, we also examined dutasteride, a broader spectrum 5AR inhibitor [42]. We found that 1 μM dutasteride, akin to finasteride, had no effect on LTP when administered alone (142.3 ± 11.2%, *N* = 5, *p* = 0.63 vs. control LTP; Supplemental Fig. 3), but, unlike finasteride, dutasteride completely inhibited the effects of 10 μM PRE-084 on LPS (95.4 ± 5.3%, *N* = 5, *p* < 0.005 vs. PRE-084 + LPS; Fig. 4C).

As shown in Fig. 2, 1 μM PRE-084 appeared to have a weak, but non-significant interaction with LPS effects while 10 μM had a very strong effect that might interfere with reversal by finasteride. This prompted us to examine the effect of finasteride on an intermediate PRE-084 concentration. We found that 3 μM, like 10 μM PRE-084 also prevented the effects of LPS on LTP (144.9 ± 14.4%, *N* = 5; *p* < 0.02 vs. LPS, Fig. 4D). Unlike 10 μM PRE-084, however, effects at 3 μM were prevented by finasteride (94.0 ± 4.5%, *N* = 5; *p* < 0.01 vs. PRE-084 + LPS, Fig. 4D). These results indicate that the two SSRIs and the S1R agonist promote the production of 5α-reduced steroids as at least one major contributor to their mechanism in preventing effects of acute LPS on hippocampal plasticity.

Sertraline is a structurally distinct, high-affinity SSRI [22] that also binds S1Rs with relatively high affinity, but unlike fluvoxamine and fluoxetine exhibits antagonist or inverse agonist effects on S1Rs in functional assays [8, 23]. At 1 μM, sertraline had no effect on baseline transmission in CA1, but in contrast to fluvoxamine and fluoxetine, inhibited LTP when administered alone (97.3 ± 5.5%, *N* = 5, *p* < 0.005 vs. control LTP; Fig. 5A). LTP inhibition by sertraline was reversed by the selective S1R agonist, 1 μM PRE-084 (131.9 ± 11.3%, *N* = 5, *p* < 0.05 vs. sertraline alone; Fig. 5A). LTP inhibition by sertraline is distinct from the effects of the more selective S1R antagonist NE-100, which had no effect on LTP induction alone (Supplemental Fig. 2). Because prior studies indicate that sertraline can act as an S1R inverse agonist in some assays, exhibiting actions that are the opposite of S1R agonizts and blocked by NE-100 [8, 23], we also examined sertraline in the presence of NE-100. Akin to what we observed with PRE-084, we found that the complete LTP inhibition by sertraline alone was at least partially reversed by 1 μM NE-100 (123.4 ± 2.9%, *N* = 7, *p* < 0.005 vs. sertraline alone; Fig. 5B), supporting a key role of S1Rs in the ability of sertraline to inhibit LTP. We also examined the combination of sertraline and 1 μM PRE-084 against LPS-mediated LTP inhibition and found variable but significant recovery of LTP compared to LPS alone (124.3 ± 7.5%, *N* = 8, *p* < 0.05 vs. LPS alone; Fig. 5C). Thus, sertraline differs from fluvoxamine and fluoxetine and likely acts as an S1R inverse agonist in the CA1 region.

**Fig. 6:**
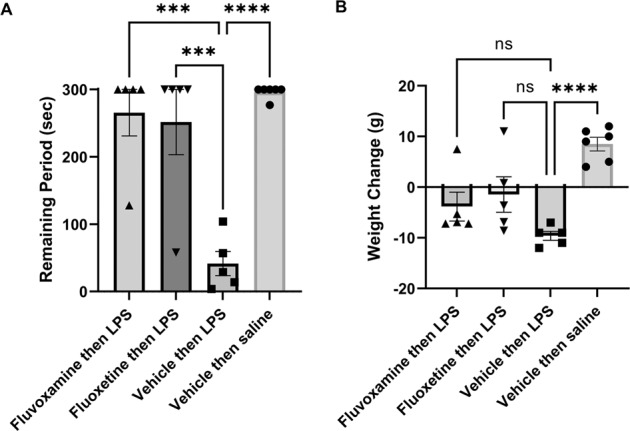
Fluvoxamine and fluoxetine prevent memory impairment by LPS. **A** The graph depicts the time that animals remained in the lit chamber on the day following conditioning during a 5 min trial. When administered 1 h prior to LPS followed by conditioning in a one-trial inhibitory avoidance task, both fluvoxamine (10 mg/kg i.p.) and fluoxetine (10 mg/kg i.p.) prevented the adverse effects of LPS on learning. When LPS (1 mg/kg i.p.) was administered 1 h following vehicle (DMSO) and 1 h prior to conditioning, rats were markedly impaired in one-trial learning. Animals receiving vehicles followed one hour later by saline (the vehicle for LPS), showed intact learning. Statistical analyses show results from Dunnett’s multiple comparison tests (***p = 0.0002; ****p < 0.0001). **B** Twenty-four hours after LPS, rats showed weight loss, whereas vehicle-treated controls showed weight gain. Neither fluvoxamine nor fluoxetine altered LPS-induced weight loss by Dunnett’s test (****p < 0.0001).

To determine whether observations in hippocampal slices translate to changes in behavior, we examined the effects of fluvoxamine and fluoxetine using a one-trial inhibitory avoidance learning task that has previously been linked to CA1 hippocampal LTP [34, 35, 43] and we have shown is disrupted by acute treatment with LPS [31]. One day following aversive (shock) conditioning in the dark chamber of the two-chamber apparatus, control rats treated with vehicle alone (DMSO followed by saline) remained in the lit chamber for nearly the entire 300 s test period (296.2 ± 3.8 s, *N* = 6; Fig. 6A), indicating the establishment of memory for the shock. In contrast, rats receiving vehicle one hour prior to LPS showed markedly impaired memory one day after conditioning, remaining in the safe (lit) chamber for only 41.6 ± 18.0 s, *N* = 5, *p* < 0.0001 vs. vehicle alone) of the 300 s trial.

Both fluvoxamine and fluoxetine pretreatment prevented acute LPS-induced memory impairment. Animals pretreated with fluvoxamine (10 mg/kg i.p. one hour prior to LPS) remained in the lit compartment for 265.6 ± 34.4 s (*N* = 5; *p* < 0.001 vs. LPS), while those treated with fluoxetine remained in the light for 251.6 ± 48.4 s (*N* = 5) of the 300 s trial (*p* < 0.001 vs. LPS; Fig. 6A). As expected, rats treated with LPS lost weight over the ensuing 24 h compared to vehicle-treated controls (LPS: -9.6 ± 0.9 g, *N* = 6 vs. vehicle controls: +8.5 ± 1.3 g, *N* = 5; *p* < 0.0001; Fig. 6B). LPS-induced weight loss was not altered significantly by either fluoxetine (−1.5 ± 3.5 g, *N* = 5) or fluvoxamine (−3.8 ± 2.4 g, *N* = 5; Fig. 6B).

## Discussion

Evidence that fluvoxamine may prevent deterioration in individuals with COVID-19 [1, 27–29], but see [44], along with observational data suggesting that other SSRIs may share effects and mechanisms with fluvoxamine [45], have spurred interest in understanding how SSRIs alter inflammatory states. A leading hypothesis that prompted the initial fluvoxamine COVID-19 clinical trial was based on agonist actions at S1Rs as a mechanism to dampen excessive inflammatory reactions [1, 2], a notion supported by preclinical studies [26]. In the present study, we examined the effects of fluvoxamine and two other SSRIs, fluoxetine and sertraline, in a well-characterized model of neuroinflammation that results in the disruption of synaptic plasticity in ex vivo rat hippocampal slices [31]. For these studies, we used pretreatment with SSRIs before delivery of pro-inflammatory stimulation to determine whether such treatment could prevent deterioration in hippocampal function; observational clinical data also suggest the benefits of SSRIs administered prior to COVID diagnosis [45].

Consistent with our recent observations [30], brief (15 min) exposure to low μg/ml concentrations of LPS, a cell wall endotoxin from gram-negative bacteria and a known pro-inflammatory stimulus, disrupts induction of CA1 hippocampal LTP and learning without altering basal transmission. This form of LPS treatment produces acute and strong inflammatory activation; effects on LTP are mimicked by lower (ng/ml) concentrations of LPS applied for 2–4 h or more [30]. We found that pretreatment with fluvoxamine, at a concentration achieved with clinical dosing [24], prevented the effects of LPS on LTP induction; fluvoxamine also prevented learning defects induced by LPS in vivo. While effective brain concentrations of SSRIs are not certain, available evidence indicates they achieve low micromolar levels consistent with concentrations used in our experiments [4, 24, 46]. A selective S1R antagonist blocked the protective effects of fluvoxamine on LTP, strongly supporting the hypothesis that S1R agonism is a key mechanism underlying this anti-inflammatory action. Consistent with this observation, a selective S1R agonist also prevented the effects of LPS on LTP.

A different SSRI, fluoxetine, also prevented the effects of LPS on LTP and learning. However, complete inhibition of LPS required a higher concentration than fluvoxamine, but a concentration is still consistent with drug levels achieved with clinical dosing [4, 24, 46]. Initially, we hypothesized that the need for higher fluoxetine concentration reflected the lower potency of this SSRI at S1Rs [8, 19]. Unlike fluvoxamine, however, a selective S1R antagonist failed to overcome the effects of fluoxetine on LPS-mediated LTP inhibition, at a concentration of the antagonist that completely blocked the effects of fluvoxamine and the selective S1R agonist, PRE-084. This prompted us to examine other mechanisms by which fluoxetine may alter the effects of LPS. Based on prior studies indicating that fluoxetine and other SSRIs promote endogenous neurosteroid synthesis [13, 38] and previously described anti-inflammatory actions of certain neurosteroids [47, 48], we examined the effects of 5AR inhibitors, which prevent the synthesis of 5α-reduced neurosteroids including allopregnanolone. In the presence of finasteride, the ability of both fluoxetine and fluvoxamine to prevent LPS-induced LTP inhibition was blocked, indicating an important role for neurosteroids in anti-inflammatory actions.

The effects of the selective S1R agonist PRE-084 also involve neurosteroids but show important differences from the SSRIs because finasteride had no effect on the ability of 10 μM PRE-084 to overcome LTP block by LPS. In contrast, dutasteride, a broader spectrum 5AR antagonist [42, 49], prevented the effects of this concentration of PRE-084 on LPS. We also observed, however, that a lower concentration of PRE-084 (3 μM) blocked the effects of LPS in a finasteride-sensitive fashion. Both Type I and Type II 5ARs are expressed in the hippocampus [40, 41]. Type II 5AR is inhibited more potently by finasteride in a mechanistically distinct way from Type I 5AR [50] and promotes neurosteroid production under conditions of low substrate availability. In contrast, Type I 5AR, which is inhibited potently by dutasteride, is active at higher concentrations of steroid precursors [51]. Taken together the present studies indicate an important role of S1Rs in promoting neurosteroidogenesis [11], and 5α-reduced neurosteroids play a key role in the modulatory effects of S1R agonism on pro-inflammatory changes in plasticity.

Both S1R agonism and neurosteroids are involved in the actions of fluvoxamine. In contrast, fluoxetine’s effects on LPS-induced effects on LTP do not appear to involve S1Rs, and this SSRI has other mechanisms that promote neurosteroid synthesis, including modulation of 3α-hydroxysteroid dehydrogenase (3α-HSD), a key enzyme in neurosteroid synthesis [10], but see [52]. Intriguingly, neurosteroids are important endogenous modulators of neuronal stress and our present studies indicate that endogenous 5α-reduced neurosteroids promote hippocampal plasticity (and learning) under the stress of pro-inflammatory stimulation. This plasticity-enhancing effect stands in contrast to the previously described ability of 5α-neurosteroids to dampen LTP induction under other stressful conditions [43]. Negative effects on LTP are mediated, at least in part, by positive allosteric modulation of GABA-A receptors; mechanisms and conditions contributing to the enhancement of plasticity by 5α-neurosteroids are uncertain but could involve known intracellular actions of these steroids including effects on autophagy and pro-inflammatory signaling [47, 48, 53–55].

In contrast to fluvoxamine and fluoxetine, low micromolar sertraline, another high-potency SSRI [22], had no effect on baseline CA1 transmission but inhibited LTP in the absence of LPS. This observation makes it unlikely that inhibition of serotonin transport alone is the primary driver of the effects of SSRIs against LPS. Sertraline binds S1Rs with a potency that is intermediate between fluvoxamine and fluoxetine but differs from these other SSRIs in functioning as an apparent S1R antagonist or inverse agonist [8, 22, 23, 56]. In a neurite outgrowth assay, sertraline has effects that are opposite of fluvoxamine, fluoxetine, and PRE-084, and differs from the S1R antagonist, NE-100 [8, 56]. Sertraline also antagonizes the effects of the other two SSRIs, akin to NE-100. Effects of sertraline in the neurite extension assay are prevented by both an S1R agonist and an S1R antagonist [8, 56], consistent with inverse agonism by sertraline. In our studies, the inhibitory effects of sertraline on LTP induction were overcome by an S1R agonist (PRE-084), even though a more selective and pure S1R antagonist (NE-100) had no effect on LTP by itself. Additionally, NE-100 at least partially overcame LTP inhibition by sertraline, strongly suggesting that sertraline functions as an S1R inverse agonist in our LTP assay. A combination of sertraline with the S1R agonist also partially overcame the effects of LPS on LTP. Taken together, these results indicate that the effects of sertraline on neuronal plasticity are complex and include actions at S1Rs.

While our results are consistent with the importance of S1Rs in contributing to the effects of fluvoxamine, it is also clear that SSRIs have multiple other actions that could contribute to the anti-inflammatory effects we observed [2]. Beyond well-known effects on serotonin, inhibition of acid sphingomyelinase (ASM) and lysosomal effects could contribute given that ASM inhibition can trigger the cellular process of autophagy, providing a mechanism to dampen cellular stress, modulate inflammation and promote neuroplasticity [9]. Additionally, fluvoxamine inhibits cellular stress responses as a mechanism to dampen inflammation [12, 26], and fluoxetine and fluvoxamine can directly inhibit the NLRP3 inflammasome [3]

Our results support the hypothesis that S1R agonism contributes to the ability of fluvoxamine to dampen the adverse effects of a pro-inflammatory stimulus on hippocampal function. While SSRIs are known to have anti-inflammatory effects that could involve several cellular mechanisms [57], our results with PRE-084 (Figs. 2 and 4), indicate that activation of S1Rs independent of SSRI activity is an important mechanism that contributes significantly to the effects of fluvoxamine, but not fluoxetine. S1Rs are important regulators of multiple cellular processes in endoplasmic reticula (ER), mitochondria, nuclei, and synapses. These receptors are enriched in ER–mitochondrial-associated membranes where they serve as ligand-operated molecular chaperones [21] that help to regulate ER and mitochondrial stress [17]. Under basal conditions, S1Rs bind BiP (binding immunoglobulin protein, also called GRP78) and are in an inactive state. Upon agonist binding, BiP dissociates from S1Rs allowing translocation of the receptor to various membranes and interactions with other proteins that include mitochondrial proteins such as voltage-dependent anion channel (VDAC) and proteins involved in cellular regulation and signaling including NMDARs and other ion channels [11, 22]. In cells under stress, S1R activation dampens ER stress, maintains calcium homeostasis and mitochondrial function, decreases the production of reactive oxygen species (ROS), and promotes cholesterol trafficking for steroidogenesis (including synthesis of pregnenolone, the first step in neurosteroid production) [11, 19]. As a result of these diverse actions, S1Rs are thought to play important roles in brain function including learning, memory, and cognition [22]. Interestingly, the knockdown of S1Rs impairs pregnenolone synthesis but does not alter the expression of 3α-HSD, a protein through which fluoxetine appears to regulate neurosteroid synthesis [11].

In summary, our results support the hypothesis that fluvoxamine and fluoxetine have anti-inflammatory effects that help to preserve neuronal function under acute inflammatory stress. Whether and how these effects, including effects on S1Rs and neurosteroids, contribute to psychotropic actions remains uncertain [58], but there is increasing evidence that allopregnanolone (brexanolone) and certain synthetic neuroactive steroid analogs have beneficial effects as therapeutics in psychiatric illnesses [55]. Furthermore, the ability of SSRIs to promote synaptic plasticity under stressful conditions may contribute to therapeutic actions [59–61]. Our results further suggest that certain SSRIs may have beneficial effects beyond primary psychiatric illnesses, particularly in neuropsychiatric disorders associated with neuroinflammation and cognitive dysfunction.

## Supplementary information

Supplementary Legends

Supplemental Figure 1

Supplemental Figure 2

Supplemental Figure 3

Details of statistical analyses for physiology experiments in Figures 1-5.
