# Supplementary material for: SSRIs differentially modulate the effects of pro-inflammatory stimulation on hippocampal plasticity and memory via sigma 1 receptors and neurosteroids
Source: Transl Psychiatry. 2023 Feb 3;13:39. doi: 10.1038/s41398-023-02343-3 (PMC9897619; doi:10.1038/s41398-023-02343-3)
Supplement: Supplementary file 1 — Supplementary Legends [file 41398_2023_2343_MOESM1_ESM.docx]

**SUPPLEMENTAL FIGURE AND TABLE LEGENDS**

**Supplemental Figure 1.** Fluvoxamine and fluoxetine alone do not alter LTP in naïve slices. When administered at 1μM, prior to and during HFS, neither fluvoxamine (A) nor fluoxetine (B) altered LTP induction. Traces show representative EPSPs as in other figures.

**Supplemental Figure 2**. Effects of S1R ligands on LTP induction. A. NE-100, a selective S1R antagonist, does not alter LTP when administered alone. B. Similarly, NE-100 did not alter LTP induction in the presence of fluvoxamine. C. PRE-084, a selective S1R agonist, did not alter LTP when administered alone. Traces show representative EPSP. Calibration: 1 mv, 5 ms.

**Supplemental Figure 3**. 5AR inhibitors do not alter LTP in control slices. A. In the presence of 1 μM finasteride, HFS readily induced LTP in naïve slices. B. LTP is also induced in the presence of 1 μM dutasteride. Traces show EPSPs as in Figure 1. Calibration 1 mV, 5 ms.

**Supplemental Table 1**. Details of statistical analyses for physiology experiments in Figures 1-5. Abbreviations: U t-test (unpaired t-test); Mean Diff (Mean difference between samples); 95% CI (95% confidence interval); Fin (finasteride); Dut (dutasteride); Sert (sertraline); Sert + Pre (sertraline + PRE-084).
