# Supplementary figures and images for: SSRIs differentially modulate the effects of pro-inflammatory stimulation on hippocampal plasticity and memory via sigma 1 receptors and neurosteroids
Source: Transl Psychiatry. 2023 Feb 3;13:39. doi: 10.1038/s41398-023-02343-3 (PMC9897619; doi:10.1038/s41398-023-02343-3)

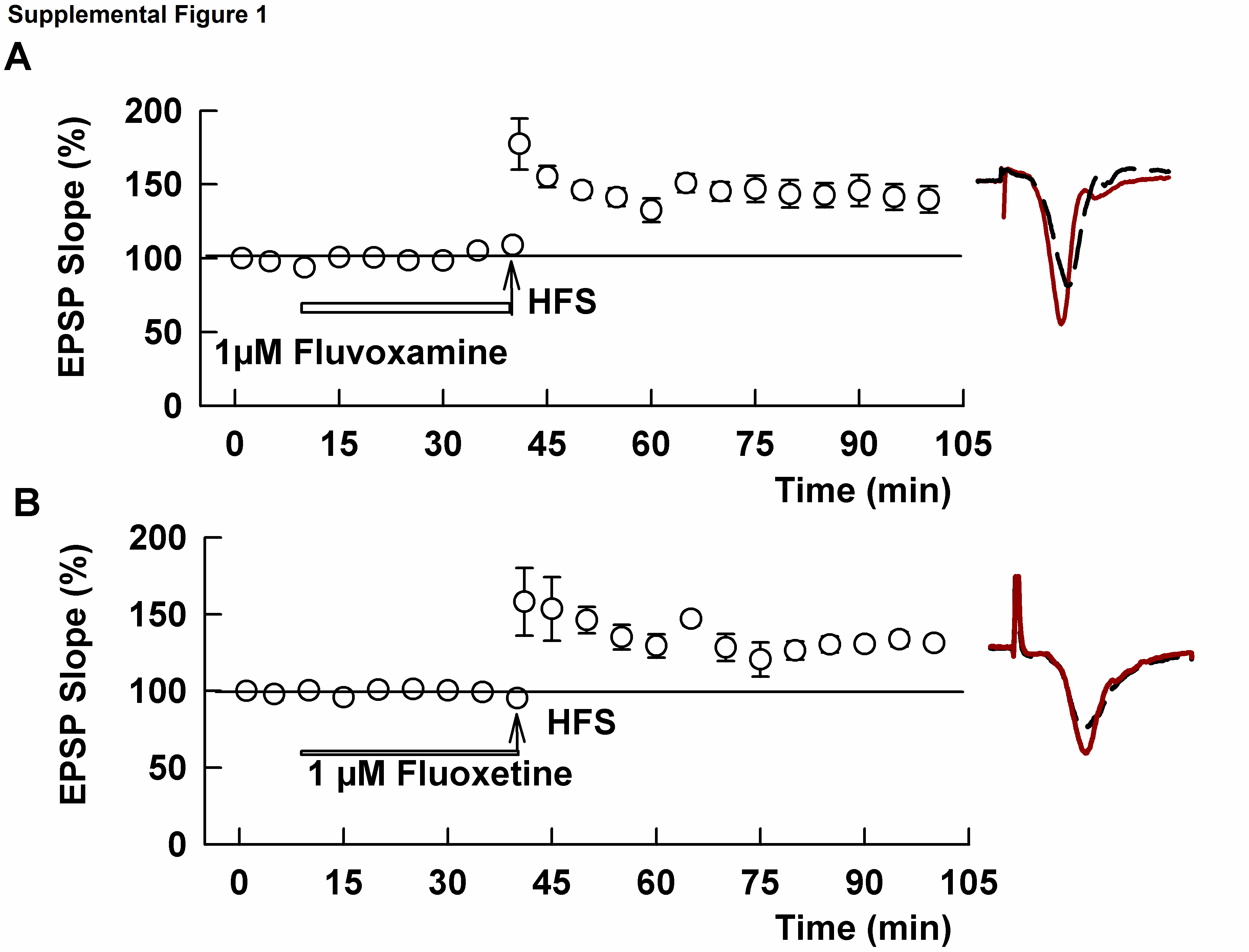

Supplement: Supplementary file 2 — Supplemental Figure 1 [file 41398_2023_2343_MOESM2_ESM.tif]

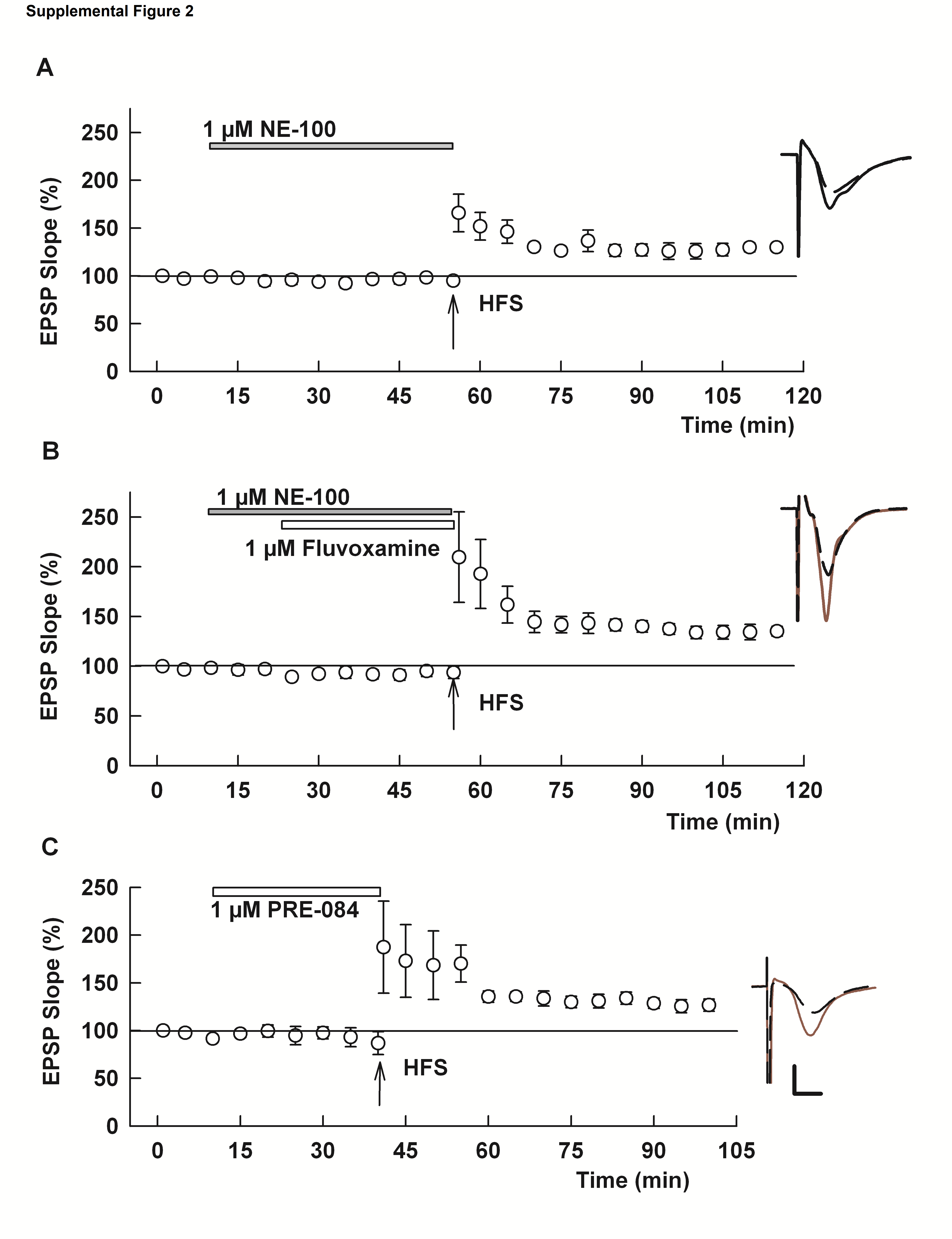

Supplement: Supplementary file 3 — Supplemental Figure 2 [file 41398_2023_2343_MOESM3_ESM.tif]

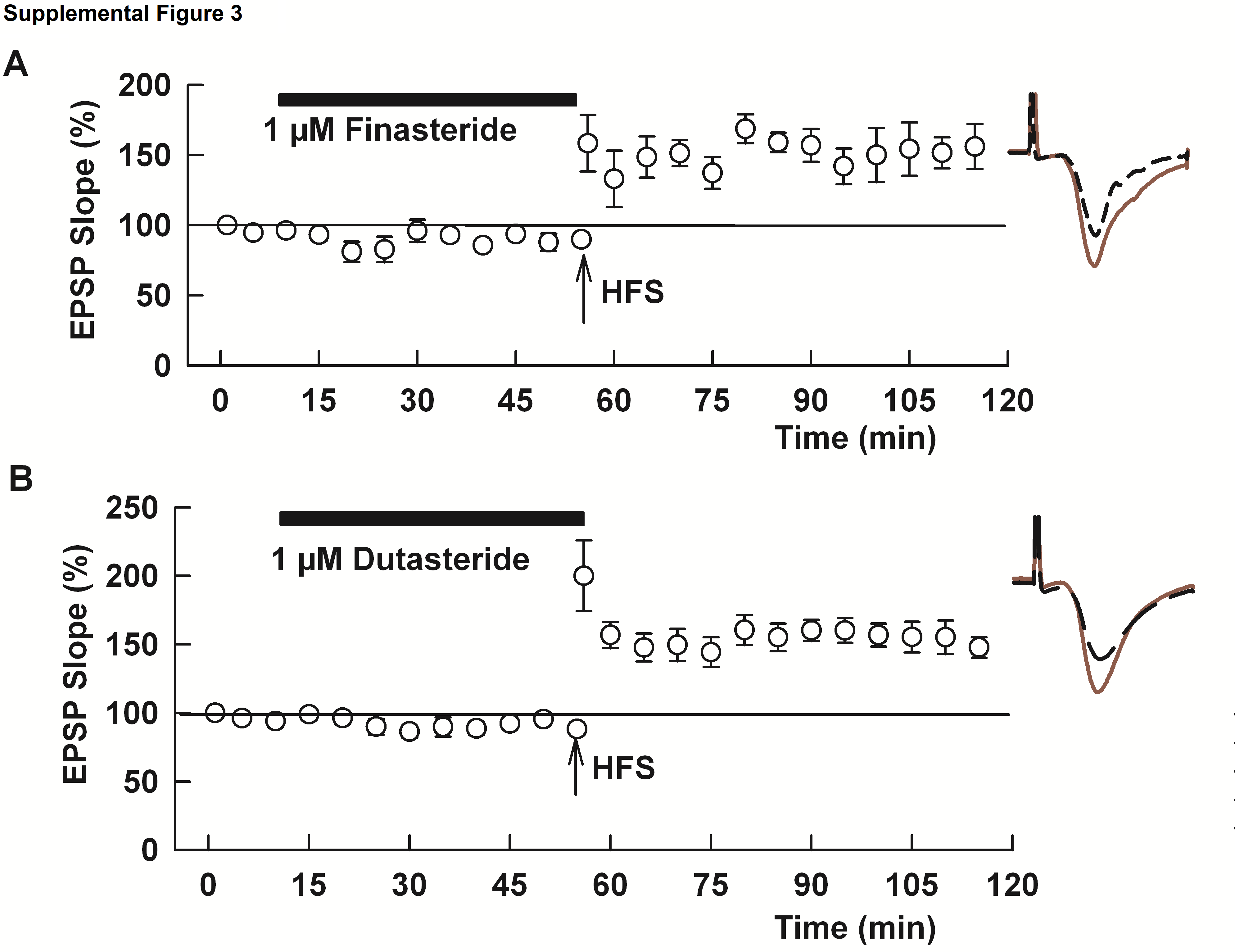

Supplement: Supplementary file 4 — Supplemental Figure 3 [file 41398_2023_2343_MOESM4_ESM.tif]
