# Supplementary material for: SSRIs differentially modulate the effects of pro-inflammatory stimulation on hippocampal plasticity and memory via sigma 1 receptors and neurosteroids
Source: Transl Psychiatry. 2023 Feb 3;13:39. doi: 10.1038/s41398-023-02343-3 (PMC9897619; doi:10.1038/s41398-023-02343-3)
Supplement: Supplementary file 5 — Details of statistical analyses for physiology experiments in Figures 1-5. [file 41398_2023_2343_MOESM5_ESM.docx]

**Supplemental Table 1 – Statistical Analyses**

| Figure | Test | Mean Diff | 95% CI | t-score | Hedge’s g |
| --- | --- | --- | --- | --- | --- |
| 1A | U t-test | 38.4 | 21.4-55.4 | 5.21 | 3.29 |
| 1B | U t-test | 51.2 | 23.1-79.3 | 4.12 | 2.49 |
| 1C | U t-test | 51.1 | 22.3-79.9 | 4.01 | 2.42 |
| 2A | U t-test | 17.1 | -0.6-34.8 | 2.19 | 1.33 |
| 2B | U t-test | 50.1 | 24.7-75.5 | 4.55 | 2.88 |
| 2C | U t-test | 55.0 | 31.2-78.7 | 5.33 | 3.37 |
| 3A | U t-test | 28.8 | -10.3-67.9 | 1.70 | 1.07 |
| 3B | U t-test | 74.2 | 46.8-101.6 | 6.23 | 3.94 |
| 3C | U t-test | 52.4 | 18.8-86.0 | 3.59 | 2.27 |
| 3D | U t-test | -5.9 | -41.6-29.8 | 0.38 | 2.41 |
| 4A | U t-test | 60.7 | 26.4-95.0 | 4.09 | 2.58 |
| 4B | U t-test | 55.2 | 24.9-85.5 | 4.20 | 2.58 |
| 4C Fin | U t-test | -6.5 | -35.8-22.8 | 0.51 | 0.32 |
| 4C Dut | U t-test | 52.3 | 26.3-78.3 | 4.64 | 2.93 |
| 4D | U t-test | 50.9 | 16.1-85.7 | 3.38 | 2.13 |
| 5A Sert | U t-test | 38.7 | 20.3-57.1 | 4.86 | 3.07 |
| 5A Sert+Pre | U t-test | 34.6 | 5.5-63.7 | 2.74 | 1.73 |
| 5B | U t-test | 26.1 | 13.2-39.0 | 4.52 | 2.65 |
| 5C | U t-test | 30.1 | 0.4-59.8 | 2.33 | 1.48 |
